# Physiology and Inflammation Driven Pathophysiology of Iron Homeostasis—Mechanistic Insights into Anemia of Inflammation and Its Treatment

**DOI:** 10.3390/nu13113732

**Published:** 2021-10-22

**Authors:** Lukas Lanser, Dietmar Fuchs, Katharina Kurz, Günter Weiss

**Affiliations:** 1 Department of Internal Medicine II, Medical University of Innsbruck, 6020 Innsbruck, Austria; lukas.lanser@i-med.ac.at (L.L.); katharina.kurz@i-med.ac.at (K.K.); 2 Division of Biological Chemistry, Biocenter, Medical University of Innsbruck, 6020 Innsbruck, Austria; dietmar.fuchs@i-med.ac.at; 3 Christian Doppler Laboratory for Iron Metabolism and Anemia Research, Medical University of Innsbruck, 6020 Innsbruck, Austria

**Keywords:** anemia of chronic disease, anemia of inflammation, iron, hepcidin, erythropoietin, infection, cancer, chronic kidney disease, chronic heart failure, auto-immune disease, macrophage

## Abstract

Anemia is very common in patients with inflammatory disorders. Its prevalence is associated with severity of the underlying disease, and it negatively affects quality of life and cardio-vascular performance of patients. Anemia of inflammation (AI) is caused by disturbances of iron metabolism resulting in iron retention within macrophages, a reduced erythrocyte half-life, and cytokine mediated inhibition of erythropoietin function and erythroid progenitor cell differentiation. AI is mostly mild to moderate, normochromic and normocytic, and characterized by low circulating iron, but normal and increased levels of the storage protein ferritin and the iron hormone hepcidin. The primary therapeutic approach for AI is treatment of the underlying inflammatory disease which mostly results in normalization of hemoglobin levels over time unless other pathologies such as vitamin deficiencies, true iron deficiency on the basis of bleeding episodes, or renal insufficiency are present. If the underlying disease and/or anemia are not resolved, iron supplementation therapy and/or treatment with erythropoietin stimulating agents may be considered whereas blood transfusions are an emergency treatment for life-threatening anemia. New treatments with hepcidin-modifying strategies and stabilizers of hypoxia inducible factors emerge but their therapeutic efficacy for treatment of AI in ill patients needs to be evaluated in clinical trials.

## 1. Introduction

Anemia is common in patients with acute or chronic inflammatory disorders, and termed as anemia of chronic disease (ACD) or anemia of inflammation (AI) [1,2,3,4]. AI is the most frequent anemia etiology in hospitalized patients and suggested to be the second most prevalent anemia after iron deficiency anemia (IDA), accounting for up to 40% of all anemias worldwide [5]. In patients with AI, disturbances of iron metabolism, dysfunction of erythropoietin, suppression of erythropoiesis and reduction of erythrocyte survival are caused by an activated immune system [2]. Patients with AI typically present with a mild to moderate, normochromic, normocytic anemia accompanied by reduced serum iron and normal to elevated iron stores [1].

Initially linked to infectious diseases, cancer or autoimmune disorders [6,7,8,9,10,11], AI is also common in patients with chronic kidney disease (CKD) [12,13,14,15], coronary artery disease (CAD) [16], chronic heart failure (CHF) [17,18], advanced atherosclerosis [19] or chronic pulmonary disease [20,21,22]. Its occurrence is associated with progression and severity of the underlying disease, decreased quality of life (QoL), a reduced cardio-vascular performance and an adverse outcome [6,7,8,9,10,11,12,13,14,15,16,17,18,19,20,21,22,23,24,25,26]. In cancer patients for instance, anemia is a common comorbidity found in approximately 40–60% of patients with malignancies mostly due to AI but often aggravated by anti-cancer therapy [27,28]. It is associated with fatigue and poor QoL [25,29] as well as impaired local tumor control [30,31]. Moreover, anemia is encountered in approximately 20% of CHF patients [32] and associated with higher NYHA classes, increased NT-proBNP concentrations and mortality [17]. Whether anemia deteriorates the underlying disease or only reflects an advanced disease stage is still controversial. Anemia in the elderly is also suggested to be partly caused by an activated immune system and thus AI shares several features with other pathologies [33,34].

This review will first summarize the physiology of iron metabolism and erythropoiesis, as well as pathophysiology and diagnosis of AI and then focus on established therapies and new therapeutic options, which are currently evaluated in clinical or laboratory studies in patients with anemia and inflammatory disease.

## 2. Iron Homeostasis and Erythropoiesis

Iron (Fe) is crucially involved in multiple important biochemical pathways, including mitochondrial respiration, metabolic processes, hormone synthesis or deoxyribonucleic acid (DNA) synthesis (reviewed by Muckenthaler et al. [35]). However, its best-known role is the one as the functional component of hemoglobin and myoglobin to enable oxygen binding and transport. At least two million erythrocytes are normally synthesized per second in the adult human bone marrow. Each of these erythrocytes contains about 280 million hemoglobin molecules, which in turn contain four heme-subunits with central iron atoms. These iron atoms represent the binding sites for oxygen thus providing more than a billion oxygen binding sites per erythrocyte. [36,37] The daily iron demand for metabolic processes in adult humans is about 20–30 mg. Most of this daily required iron comes from macrophages, which recycle iron by phagocytosis of senescent erythrocytes, while about 10% of the daily needs are provided by duodenal absorption of iron from the diet. Iron is found in both vegetables and meat, with heme bound iron having a higher bioavailability [38,39]. Iron can also catalyze the generation of radicals which damage cellular macromolecules thus promoting cellular death and tissue damage [40,41]. These reactive radicals can have favorable anti-microbial effects, but usually are causing tissue injury and organ failure as seen in patients with hemochromatosis [42,43]. Therefore, iron homeostasis needs to be tightly regulated in the human body.

### 2.1. Duodenal Iron Absorption, Circulatory Iron Transport and Cellular Iron Uptake

Iron is absorbed in proximal duodenal endothelial cells either as heme iron via the heme transporter heme carrier protein 1 (HCP1) or as non-heme iron via the divalent metal transporter 1 (DMT1). Luminal non-heme iron (principally Fe^3+^ complex formations) is first reduced by the duodenal cytochrome B (DCYTB) to Fe^2+^ before it is transported across the cellular membrane by DMT1 [44,45]. Iron is either stored as mucosal ferritin in duodenal endothelial cells or transported to the bloodstream by ferroportin 1 (FPN1) [46]. FPN1 is the only known cellular iron exporter and not only responsible for iron export from duodenal enterocytes but also for iron mobilization from hepatocytes and erythrocyte recycling macrophages [37,47]. After Fe^2+^ is exported by FPN1, it is oxidized to Fe^3+^ by hephaestin (HEPH) at the basolateral membrane of duodenal enterocytes and attached to transferrin [44]. Contrary, mucosal ferritin is lost through shedding of mucosal endothelial cells over time. This is the only route for body iron elimination since there is no regulated iron excretion. [48]

Iron transport in the circulation occurs almost entirely bound to the beta globulin transferrin, which is synthesized in the liver [49]. Iron also binds to lactoferrin, which further regulates the proliferation and activation of monocytes/macrophages, lymphocytes and natural killer cells (NK-cells) [50]. Cells requiring iron express a transferrin receptor (TFR) in their membrane: TFR1 is ubiquitously expressed while TFR2 is only expressed in hepatocytes, duodenal crypt cells, erythroid cells, osteoblasts, neurons and macrophages [51,52]. Moreover, TFR1 is post-transcriptionally regulated by iron via iron regulatory proteins (IRP), while TFR2 is not regulated by IRPs [53,54]. Transferrin, when loaded with two molecules of iron, has an approximately 30-fold higher affinity to TFR1 than to TFR2 [55]. The transferrin-Fe-TFR1 complex is internalized by cells through endocytosis after transferrin has bound to TFR1, while TFR2 has a regulatory function unrelated to iron transport [56,57]. After the endocytic vesicle with the transferrin-Fe-TFR1 complex coalesces with a primary lysosome, Fe^3+^ is released from transferrin in the acid lysosomal milieu and converted via the action of six transmembrane epithelial antigen of the prostate 3 (STEAP3) to Fe^2+^ [58] and then transported to the cytosol by DMT1 [59]. Transferrin-Fe binding to TFR2 activates among others ERK1/2 and p38 MAPK pathway [60,61] and further modulates BMP/SMAD pathway (see below) [62].

Most of the iron is stored in hepatocytes and macrophages of the reticuloendothelial system bound to ferritin [63]. Iron stores in humans can usually sustain iron requirements for several years. While hepatocytes acquire iron primarily by transferrin uptake, macrophages retain iron from uptake and digestion of senescent erythrocytes, also named erythrophagocytosis [63]. Hemosiderin, another iron storage protein, is formed when ferritin is partially digested and deposited at lysosomal membranes [64]. It is primarily found in cells with iron overload and mobilizes iron irregularly and slowly [65,66]. Finally, iron can be mobilized from ferritin via nuclear receptor coactivator 4 (NCOA-4) mediated autophagy [67] and then exported from cells by FPN1 [68]. After excretion of Fe^2+^ by FPN1, in hepatocytes and macrophages the ferroxidase ceruloplasmin oxidizes Fe^2+^ to Fe^3+^, which is then again bound to transferrin [68,69]. In addition, ferritin can be secreted by vesicular pathways to the circulation [70].

### 2.2. Systemic Regulation of Iron Metabolism

Hepcidin is the main systemic regulating hormone of iron metabolism and is primarily synthesized in the liver [71,72]. Hepcidin binds to FPN1, which induces its lysosomal degradation [69]. This reduces circulating iron concentrations by decreasing release of recycled iron from macrophages and stored iron in hepatocytes concomitant to a reduced intestinal iron uptake. [71,73]. Bone morphogenetic protein 6 (BMP6), a member of the tumor growth factor beta (TGF-β) family, is the most potent inducer of hepcidin production and it is expressed in the liver in response to iron overload [62,74,75]. It binds to BMP receptor type 1 (BMPR1) in conjunction with the co-receptor hemojuvelin (HJV) thus upregulating *HAMP* expression via SMAD1/5/8 signaling [76,77]. More recently, BMP2 was also shown to be critically involved in *HAMP* expression. It is regulated by dietary iron (although to a lesser extent than BMP6) and binds to HJV with a similar high affinity as BMP6 [78,79]. BMP2/6 preferentially signals via the BMPR1 activin receptor-like kinase 3 (ALK3) for hepcidin induction [80]. BMP2/6 regulation of HAMP gene expression represents a feedback loop to prevent iron overload with consecutive tissue damage.

**Figure 1:**
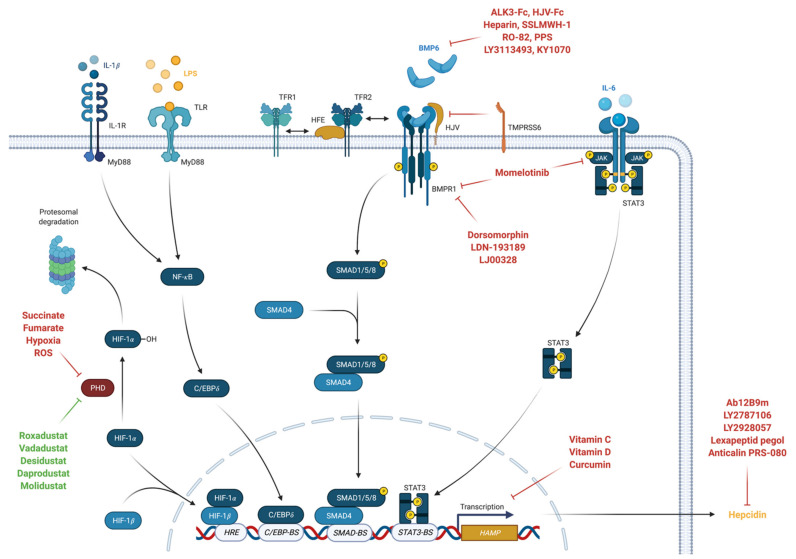
Regulation of HAMP gene expression (created with BioRender).

BMP/SMAD pathway signaling can be modulated by several iron proteins including human hemochromatosis protein (HFE), TFR2 and TMPRSS6 [62]. High concentrations of transferrin-bound iron (TFBI) competitively binds to TFR1 subsequently causing displacement of HFE which then associates with TFR2 [81]. This complex then interacts with HJV and ALK3 thus preventing its degradation, increasing ALK3 expression at the cell surface and inducing *HAMP* expression [82,83,84]. Contrary, the serine protease matriptase-2 encoded by the *TMPRSS6* gene was shown to inhibit the BMP/SMAD pathway related *HAMP* expression [85]. Because of sex differences found in iron loading disease, several studies investigated the impact of sex hormones on hepcidin regulation [62]. The male sex hormone testosterone was shown to suppress hepcidin production by activating epidermal growth factor receptor (EGFR) and by inhibiting SMAD1/5/8 signaling pathway [86,87]. Actually, testosterone administration was demonstrated to suppress serum hepcidin levels dose-dependent in men with an increase in hemoglobin levels [88]. Contrary, the female sex hormone progesterone was reported to induce hepcidin production independent of BMP/SMAD signaling [89], while data on estrogen are contradictory [90,91]. Interestingly, the recently discovered regulator of phosphate and mineral metabolism, namely fibroblast growth factor 23 (FGF23), was shown to decrease hepcidin expression induced by BMP6 or IL-6 [92]. (Figure 1).

### 2.3. Erythropoiesis, Erythrocyte Life Cycle and Erythrophagocytosis

Erythrocytes are produced in the bone marrow following differentiations and proliferations from multipotent hematopoietic stem and progenitor cells. After seven stages of differentiations from megakaryocyte erythroid progenitor cells (MEP) to proerythroblasts (ProE) and further to orthochromatic erythroblasts (OrthoE), the nucleus is expelled, and cells develop into reticulocytes. After 1–3 days, reticulocytes are released from the bone marrow into the circulation where they develop into mature erythrocytes after additional 1–2 days. Thus, the number of circulating reticulocytes represents recent erythropoiesis and is a good indicator of bone marrow activity. In addition, the hemoglobin content of reticulocytes (CHr) provides an indirect measure of functional iron availability for erythropoiesis within the previous 3–4 days [93,94].

Erythropoiesis is dependent on vitamin B9 (folic acid), vitamin B12 (cobalamin) and iron, and mainly regulated by erythropoietin (EPO), which is produced in the kidney and to a lesser extent in the liver in response to hypoxia [95,96]. In healthy individuals a feedback loop regulates EPO synthesis to maintain production of erythrocytes equable to erythrocyte destruction to sustain sufficient tissue oxygenation [95]. Of note, EPO impacts on iron delivery for erythroid progenitors by stimulating TFR1 mediated iron uptake [97], whereas iron deficiency (ID) results in reduced expression of the erythropoietin receptor (EPOR) component SCRIBBLE and thus in reduced in EPO signaling in erythroblasts [98]. Decreased serum iron reduces plasma membrane TFR2 by its internalization and lysosomal degradation alongside with SCRIBBLE thus decreasing *HAMP* expression in the liver together with an enhanced EPO sensitivity in erythroid cells [98,99,100]. Erythroblasts further produce erythroferrone (ERFE) in response to EPO via the Janus kinase (JAK)/signaling transducer and activator of transcription 5 (STAT5) signaling pathway [101]. ERFE was shown to suppress hepcidin induction by BMP2/6 thus enhancing iron availability for erythropoiesis [80,102,103]. There are also several other factors that stimulate erythropoiesis including stem cell factor (SCF) insulin, insulin-like growth factor (ILGF), activin A and angiotensin II [93,104,105].

Erythrocytes circulate for about 100–120 days before macrophages, resident in the red pulp of the spleen or in the circulation, internalize them via phagocytosis and liberate iron from heme-subunits via the enzyme heme oxygenase 1 (HMOX1) [47,106,107]. Iron is then transported to the cytosol by DMT1 where it is either stored to ferritin or released into the circulation by FPN1 [108,109]. Interestingly, iron liberated from damaged or senescent erythrocytes first accumulates in cells of the reticuloendothelial system before it is distributed to hepatocytes within one week [63]. In the bone marrow, iron is stored within resident erythroid island macrophages which play an important role in local iron management and further provide iron for erythropoiesis [110].

### 2.4. Regulation of Iron Metabolism and Erythropoiesis on Cellular Level

Iron metabolism is primarily regulated by hypoxia inducible factors (HIF) and iron regulatory proteins (IRP). HIFs respond to decreased oxygen availability not only by adaptions in iron metabolism, but also by increasing erythropoietin expression and subsequently erythropoiesis. HIF is a transcription factor composed of an alpha and beta subunit. Under normoxic conditions, HIF-𝛼 are hydroxylated, ubiquitinated and proteolytically degraded, while HIF-𝛽 are constitutively expressed. [111] Prolyl hydroxylases (PHD) catalyze the hydroxylation of HIF-𝛼 in the presence of oxygen, iron oxyglutarate and ascorbate [112,113]. Under hypoxic conditions, HIF-𝛼 cannot be hydroxylated, thus translocates to the nucleus and binds to hypoxia responsive elements (*HRE*) as heterodimer with HIF-𝛽 [114]. *HREs* occur in several genes including *EPO*, *HAMP*, *transferrin*, *TFR1*, *TFR2*, *DMT1*, *DCYTB* or vascular endothelial growth factor (*VEGF*) [115,116,117]. There are three isoforms of HIFs: HIF-1, HIF-2 and HIF-3 [111]. While HIF-2 is the predominant HIF in the regulation of FPN1, DMT1, DYCTB, and EPO transcription [118,119,120,121], HIF-1 is predominant in the regulation of TFR, ceruloplasmin, HMOX1 and hepcidin [122,123,124,125,126].

Cellular iron homeostasis is further orchestrated via the interaction of cytoplasmic iron regulatory proteins (IRP) with messenger ribonucleic acid (mRNA) stem loop structures named iron responsive elements (IRE) [127]. IREs are found within the untranslated regions of mRNAs coding for ferritin, DMT1, TFR1, FPN1 and ALAS2, the latter being a key enzyme in the biosynthesis of heme [128]. Cellular ID increases the binding affinity of IRPs to IREs [129,130,131]. While this inhibits ferritin, FPN1 and ALAS2 expression by blockage of the translation initiation complex, it increases iron uptake by prolonging half-life of TFR1 mRNA [129,132]. Thereby, the cell increases intracellular iron availability by reducing iron storage and increasing iron uptake [133]. Beside cellular ID, other conditions including nitric oxide, hydrogen peroxide and hypoxia also regulate the affinity of IRPs to IREs [134,135,136]. Interestingly, hypoxia suppresses the expression of IRP-1, which increases the translation of HIF-2𝛼 mRNA by decreasing affinity of IRP-1 to IRE in HIF-2𝛼 mRNA [137,138].

Recently, the transcription factor nuclear factor erythroid 2-related factor 2 (NRF2) was indicated as another regulator of iron metabolism and erythropoiesis [139]. NRF2 is continuously ubiquitinated and subsequent proteasomal degraded under normoxic conditions dependent on Kelch-like erythroid cell-derived protein with CNC homology-associated protein 1 (KEAP1) [140]. Oxidative stress causes a disruption of the NRF2-KEAP1 interaction thus preventing NRF2 degradation [139]. NRF2 then translocates to the nucleus where it binds to antioxidant response elements (*ARE*) found in several genes, involved in heme generation or heme catabolism including hemoglobin subunits, heme-responsive gene 1 (*HRG1*), *HMOX1*, *BMP6*, *FPN1* or *ferritin*, thus inducing their expression [139,141,142,143,144]. NRF2 stimulated *ferritin* expression increases iron storage capacities thus decreasing labile iron pool (LIP) and iron mediated oxidative stress [143]. NRF2 also induces *FPN1* expression and reduces intracellular iron levels within macrophages [145,146]. NRF2 activation is further inhibited by the transcriptional repressor BRCA-1-associated carboxy-terminal helicase (BACH1) [147]. BACH1 acts as heme sensor since it is ubiquitylated and targeted for proteasomal degradation upon binding to heme [148]. Thus erythrophagocytosis with consequently increased heme supply increases NRF2 activity with subsequent upregulation of HMOX1 (increased iron liberation from erythrocytic heme-subunits) and FPN1 (thus increasing iron export to the circulation) [141]. Interestingly, NRF2 suppression further decreases HIF-1𝛼 depicting an interplay between these two transcription pathways [149,150].

## 3. Pathophysiology of Anemia in Inflammatory Disorders

**Figure 2:**
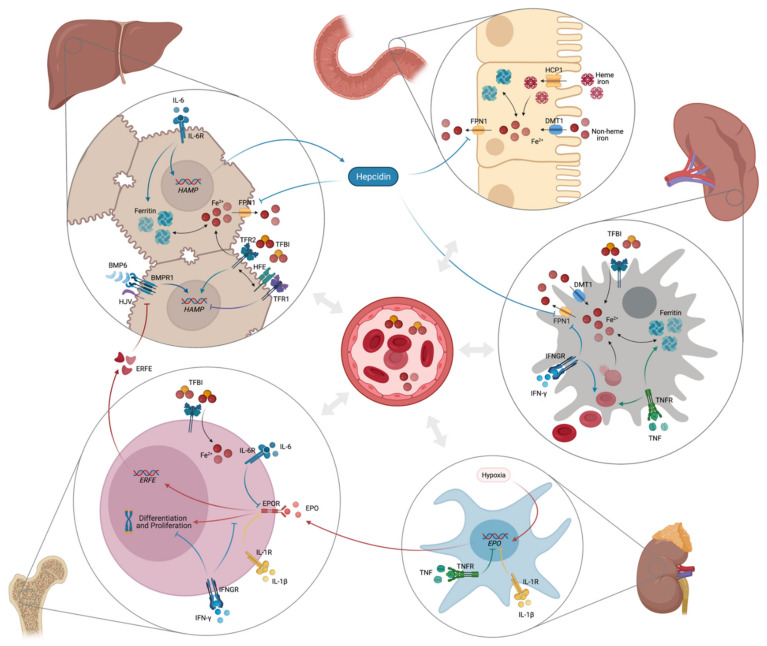
Pathophysiology of anemia of inflammation (created with BioRender).

The pathophysiology of anemia of inflammation (AI) is characterized by increased iron acquisition and retention within macrophages of the reticuloendothelial system. Concurrent intestinal iron absorption is suppressed, with consequently reduced serum iron concentrations thus limiting iron availability for erythropoiesis [1,2]. Erythropoiesis is also directly inhibited by cytokines. They damage senescent erythrocytes together with inflammation driven radicals and reduce erythrocyte half-life which is further reduced by stimulation of erythrophagocytosis [1,2,11,73,151,152,153]. Furthermore, cytokines impact on Epo formation and its biological activity thereby further contributing to anemia development [1,2,154,155]. Figure 2 gives a short overview of the main pathophysiological pathways involved in the development of AI.

### 3.1. Inflammation-Induced Disturbances of Iron Metabolism

Immune activation in response to microbes, auto-antigens or tumor antigens stimulates release of several pro-inflammatory cytokines by immune cells, which then cause disturbances in iron homeostasis [1]. Interferon gamma (IFN-γ), lipopolysaccharide (LPS) and tumor necrosis factor alpha (TNF-α) upregulate the expression of DMT1 and downregulate the expression of FPN1 with subsequent increased iron uptake and reduced iron release from pro-inflammatory macrophages of the reticuloendothelial system [37,156]. FPN1 is also expressed in duodenal enterocytes which is why under inflammatory conditions its downregulation limits also intestinal iron absorption [37]. Simultaneously, IL-1β, IL-6 but also anti-inflammatory cytokines such as IL-4 and IL-13 and IL-10 increase uptake of TFBI and upregulate synthesis of the iron storage protein ferritin by different mechanisms [1,157,158,159]. Inflammation also upregulates the expression of the iron-regulatory protein hepcidin [160]. IL-6, and to a lesser extent IL-22, stimulate *HAMP* expression in hepatocytes via the JAK/STAT3 signaling pathway [161,162]. Recent studies demonstrated that ALK3 is also involved in IL-6 mediated hepcidin response, but the exact mechanisms are still unknown [163,164]. Although inflammation suppresses expression of HJV and BMP2/6 in the liver [165,166], an intact BMP/SMAD pathway is essential for maximal inflammation-related hepcidin synthetization [62]. Activin B, a cytokine of the TNF-β family, was demonstrated to upregulate *HAMP* expression in vitro under inflammatory conditions by activating the BMP/SMAD pathway while the functional significance in vivo remains uncertain [167,168,169]. Finally, hepcidin synthesis by hepatocytes is also stimulated by IL-1β independent of BMP/SMAD or JAK/STAT3 pathway via a CCAAT enhancer-binding protein (C/EBP)-binding site (C/EBP-BS) in the *HAMP* promoter site [170]. In addition, lipopolysaccharides (LPS) via Toll-like receptor 4 (TLR4) and also IL-6 induce hepcidin expression by macrophages which then target FPN1 in an autocrine fashion [171,172].

### 3.2. Erythropoiesis Suppression and Decreased Erythrocyte Survival by Inflammation

Beside reduced iron availability for erythropoiesis, inflammation also directly impairs erythropoiesis by reducing the production and activity of EPO and repressing erythroid progenitor cells proliferation and differentiation. EPO levels are rather low in patients with AI despite hypoxia and low serum iron levels [1]. Actually, IFN-γ, IL-1β and TNF-α directly inhibit renal EPO expression [1,154]. This suppresses hypoxia-mediated EPO stimulation and induces oxidative stress thus causing harm to EPO-producing cells [173,174]. In addition, EPO-mediated signaling is impaired by these pro-inflammatory cytokines, while the number of EPOR is unchanged [174]. Reduced signal may be also linked to ID as the functional expression of the EPOR component SCRIBBLE is reduced by ID in erythroid progenitors [98]. EPO and hypoxia further inhibit *HAMP* expression by inducing HIF-1, ERFE [175], matriptase-2 [176], platelet derived growth factor BB (PDGF-BB) [177] and growth differentiation factor-15 (GDF-15) [178,179]. Inflammation-related EPO reduction thus aggravates hepcidin-mediated iron limitation to erythroid progenitor cells [2]. Moreover, several cytokines including type I interferons, IFN-γ, TNF or IL-1β have been shown to inhibit erythroid progenitor cell proliferation or differentiation directly or via induction of radical formation of ceramide mediated apoptotic processes [180,181,182]. Erythrophagocytosis by hepatic and especially splenic macrophages is also upregulated during inflammation resulting in a decreased erythrocyte half-life [2,47]. IFN-γ and TNF-α promote degradation and phagocytosis of erythrocytes thus reducing erythrocyte survival [183,184,185]. Moreover, inflammation was shown to induce erythrocyte membrane lipid remodeling and oxidative damage of erythrocytes with impaired erythrocyte function and survival [186,187].

### 3.3. Tryptophan Metabolism in the Pathogenesis of Anemia of Inflammation

IFN-*γ*, one of the main cytokines of T-helper cell type 1 (Th1) immune response, is centrally involved in the immune response to intracellular microbes and a central activator of anti-microbial immune effector pathways [188]. IFN-*γ* induces neopterin production in macrophages which reflects the extent of cellular immune activation and oxidative stress, and has thus been used as a clinical marker to monitor cellular immune activation in vivo [189]. Elevated serum concentrations of neopterin were associated with a poor outcome and anemia in patients with cancer [25,190], CAD [16], CHF [191] and pulmonary disease [192,193]. IFN-*γ* further activates indoleamine 2,3-dioxygenase (IDO) thus promoting tryptophan breakdown [194,195,196]. Tryptophan is not only essential for the growth and proliferation of various cells including hematopoietic stem and progenitor cells, but also for microbes and malignant cells. Therefore, tryptophan depletion is initially also a natural defense mechanism of the human body [197,198]. Interestingly, tryptophan metabolites including kynurenine stimulate hepcidin expression and inhibit EPO production by activating the aryl hydrocarbon receptor (AHR), which competes with HIF-2𝛼 in binding HIF-1𝛽 [199]. Moreover, quinolinic acid (QUIN), another down-stream metabolite of tryptophan, was demonstrated to inhibit EPO production [200] by preventing HIF-1𝛼 activation [201]. Tryptophan is also a crucial amino acid for brain homeostasis including serotonin and melatonin production [197]. Increased inflammation induced tryptophan breakdown via the kynurenine pathway reduces tryptophan availability for serotonin and melatonin synthesis, thus contributing to neurobehavioral symptoms including fatigue or depression [202,203], which are also common symptoms in patients suffering from anemia. Decreased tryptophan concentrations and increased kynurenine formation have been associated with decreased hemoglobin concentrations in patients with HIV-infection [204], with cancer [25,197] and also in patients with anemia of chronic disease [205]. Interestingly, both anemia and tryptophan catabolism correlated significantly with an impaired QoL and depression in patients with solid tumors [25]. Therefore, AI and tryptophan breakdown appear to be interconnected and might both contribute importantly to fatigue, depression and decreased QoL in patients with inflammatory disorders.

## 4. Diagnosis of Anemia in Patients with Inflammatory Disorders

Anemia is defined according to the criteria of the World Health Organization (WHO) as hemoglobin < 130 g/L in men and < 120 g/L in women [206]: mild anemia is defined as hemoglobin ≥ 110 g/L, moderate anemia as hemoglobin < 110 g/L and severe anemia as hemoglobin < 80 g/L. AI usually presents normocytic, normochromic, as mild to moderate anemia with decreased circulating iron concentrations and normal or increased levels of the iron storage protein ferritin while transferrin levels are reduced [155]. Because, transferrin synthesis is suppressed under inflammatory conditions and by malnutrition [155,207], transferrin saturation (TfS) may in some circumstances appear normal [208]. Therefore, the widely used threshold for TfS of 20% often fails to detect patients with pathophysiological AI as a consequence of inflammation driven Tf suppression [209,210].

The main challenge in the diagnosis is identifying the coexistence of true or absolute ID in patients with AI, because these patients need further specific diagnostics to evaluate the source of blood loss (i.e., gastrointestinal bleeding, iatrogenic blood loss due to blood sampling or other procedures, malnutrition, increased iron requirements during pregnancies or in growing children) and then different treatment as compared to subjects suffering from AI alone [181]. Several biomarkers, alone or in combination, were studied for detection of true ID in patients with ongoing inflammation. Although some studies showed promising results, these biomarkers strongly depend on laboratory equipment and were not evaluated in prospective studies [208,211,212,213]. An early and sensitive biomarker for erythropoiesis is the reticulocyte hemoglobin content (CHr), which is a reliable indicator of IDA and not influenced by inflammation [92,214]. Moreover, serum concentrations of soluble transferrin receptor (sTFR) are elevated in patients with ID reflecting an increased iron requirement of cells expressing TfR including erythroid progenitor cells [215,216]. Although sTFR was shown to be a good diagnostic biomarker for differentiating between AI with or without concomitant IDA [217], it is also affected by inflammation independent of iron status [218]. Calculation of the sTFR/log ferritin ratio, also known as ferritin index, seems to be suited well for the detection of depleted iron stores also in patients with signs of inflammation [217,219]. Currently, a ferritin index > 2 defines patients with AI and IDA, while a ferritin index < 1 defines patients with AI leaving a clinically relevant diagnostic grey zone [2,208,220]. Hepcidin plays a central role in the pathophysiology of AI, which is why its usefulness in the diagnosis of AI has been extensively studied [221,222,223]. While patients with AI have higher serum hepcidin concentrations compared to healthy controls, patients with IDA have significantly lower or even undetectable serum concentrations of hepcidin. Interestingly, patients with AI and IDA present with significantly lower hepcidin levels compared to patients with AI alone since the suppression by ID dominates over inflammation-related stimulation of hepcidin expression. [73,224,225] Nevertheless, there are still no established and clinically verified diagnostic algorithms for detecting absolute ID in patients with anemia and an activated immune system. A combination of ferritin cutoffs, transferrin and hepcidin but also of classical hematological parameters such as mean corpuscular volume (MCV) or mean hemoglobin content of erythrocytes (MCH), the latter two being reduced with ACD and true ID [155], may help in finding the right diagnosis [226].

## 5. Established Treatments of Anemia of Inflammation

The primary therapeutic approach for AI is detection and treatment of the underlying inflammatory disease, which usually results in resolution of AI over time [2]. Anti-inflammatory treatment in patients with AI based on auto-inflammatory disorders was demonstrated to ameliorate anemia in addition to an improvement of the underlying disease. TNF inhibitors including infliximab, adalimumab and certolizumab pegol play a key role in the management of inflammatory bowel disease (IBD) patients and were shown to increase hemoglobin concentrations especially in IBD patients with severe anemia [227]. Similarly, infliximab significantly improves hemoglobin levels in patients with rheumatoid arthritis [10].

More recently, the IL-1𝛽 inhibitor canakinumab reduced the prevalence of anemia and increased hemoglobin levels in anemic patients with atherosclerotic disease [228]. Accordingly, treatment with the anti-IL-6 receptor antibody toculizumab improved both, the underlying disease and anemia in patients with Castleman disease [229]. Of note, anti-IL-6R treatment of patients with advanced ovarian or lung cancer also ameliorated hemoglobin levels [230,231]. Finally, statins including simvastatin and atorvastatin downregulate hepcidin expression dose-dependently with a concomitant improvement of hemoglobin levels [232,233].

Before considering specific therapeutic options for anemia in patients with inflammatory disease, concomitant pathologies contributing to the severity of AI, including bleeding, vitamin deficiencies, hemolysis, renal insufficiency, side effects of co-medications on hematopoiesis should be evaluated and if possible treated [2,6]. If AI is not resolved by treatment of the underlying diseases or sufficient therapy of the underlying disease is not possible, other therapeutic options can be considered. The therapeutic decision must consider the underlying disease and the severity of anemia. In patients with an active infection, AI is actually a defense strategy of the body against invading microbes to restrict iron availability for pathogens also termed as “nutritional immunity” [234,235]. Therefore, treatment of AI in these patients, specifically with iron supplementation, not only promotes pathogen growth with increased virulence but also impairs the host immune response against pathogens [236,237,238,239]. There are three established therapies for the treatment of AI: iron replacement therapy, treatment with erythropoiesis-stimulating agents (ESAs) and red blood cell transfusion [2].

### 5.1. Iron Replacemant Therapy

Iron replacement therapy is an established treatment in patients with AI and should be considered, especially in patients with concomitant absolute or true ID. Iron can be supplemented either with oral or parenteral intravenous iron preparations [2]. Oral iron preparations were demonstrated to have a similarly efficacy compared to intravenous preparations in patients with inflammatory bowel disease, concomitant true ID and low disease activity [240] with the advantage of easy self-administration and low costs [241]. However, the bioavailability of oral iron supplementation is influenced by several factors. While vitamin C and overnight fasting were shown to increase iron uptake, proton pump inhibitors or milk products and tea decrease iron bioavailability [242]. Iron should be further taken only once in the morning since more frequent intake can reduce iron uptake by increased iron-related hepcidin production similar to inflammation-related hepcidin production [243]. Low absorption of oral iron increases intestinal iron concentrations, with consecutive gastrointestinal side effects including nausea, vomiting, abdominal pain and constipation, which then further limits compliance of patients [241]. Interestingly, recent studies further showed that iron therapy significantly affects the phylogenetic composition of the microbiome as well as the fecal metabolite landscape with a potential unfavorable effect on the disease [244,245]. Further studies investigating the clinical and longtime effects especially in patients with IBD are of interest, since also intravenous iron administration affects the composition of the microbiome [241].

Inflammation (even low-grade inflammation) impairs intestinal iron absorption due to hepcidin-related internalization of FPN1 making oral iron supplementation ineffective [73,246]. Therefore, parenteral intravenous iron administration is the possible option of iron supplementation in patients with chronic (inflammatory) diseases. Intravenous iron preparations have the advantage of more rapid repletion of iron deficiency. Specifically, newer carbohydrate iron formulations enable the administration of higher dosages up to 1000 mg with one infusion [241]. On the other hand, higher costs and very rare but severe anaphylactic reactions with the need for equipment to manage these life-threating situations have to be taken into consideration [247]. Although, parenteral iron administration has earlier been reserved for patients with intolerance or inadequate response to oral iron supplementation as well as for rapid iron replenishment [241], nowadays it is an established for treatment of AI with concomitant IDA in or even for ID without anemia in patients with several chronic diseases [248,249]. A meta-analysis of patients with IBD and IDA demonstrated a higher efficacy of intravenous iron administration in increasing hemoglobin levels compared to oral iron supplementation with consistently fewer gastrointestinal adverse events [250]. In CHF patients with absolute or functional ID parenteral iron supplementation was shown to improve cardiac performance and QoL even independent of concomitant anemia [251,252,253], however, concerns became evident regarding iron accumulation in the myocardium and endothelia and in regard to the need of outcome data [254]. Moreover, several clinical trials investigating iron treatment in anemic cancer patients demonstrated that intravenous (but not oral) iron administration increases hematopoietic response and decreases rate of blood transfusions with a concomitant increase of QoL [255]. However, essential endpoint data on the effect of such treatment on the course of the malignant disease are missing. Iron carbohydrate complexes are taken up by macrophages where iron is released from the carbohydrate shell and transferred to the circulation via FPN1. This transfer of iron from the macrophages is controlled by hepcidin, and it is questionable whether or not in high grade inflammation enough iron will become available for erythropoiesis, a notion which needs to be evaluated experimentally.

There are different intravenous iron formulations available which have recently been reviewed [241,256]. Although the different intravenous iron formulations are licensed and generally recommended to be used in all patients with ID, there are minor differences in physiochemical properties and recommendations by different medical societies (reviewed in [257,258]).

Despite the benefits of intravenous iron administration, there is a rare risk for serious, life-threatening hypersensitivity reactions with need for emergency response [247]. The pathogenesis of hypersensitivity reactions may differ between iron formulations and pre-existing morbidities, while the clinical presentation is the same. The two primary mechanisms of hypersensitivity reactions are immunological IgE-mediated response (e.g., to the dextran component of iron dextrans) and complement activation-related pseudo-allergy (CARPA) [259,260,261]. A recent analysis showed that the risk of severe infusion reactions is comparable between the different iron formulations and less than 1% [257,262]. Another clinical relevant side effect that was increasingly recognized lately after administration of certain intravenous formulations is hypophosphatemia [117]. Actually, moderate to severe hypophosphatemia (<2.0 mg/dL) affects up to 75.0% of patients treated with ferric carboxymaltose when compared to iron dextran (0%), ferrumoxytol (0.9%) or ferric derisomaltose (7.9%) [263,264]. Ferric carboxymaltose and to a lesser extent also ferric derisomaltose increase the expression of the hormone FGF23, which increases urinary phosphate excretion and inhibits 1-alpha hydroxylase and thus vitamin D activation [117,265]. The following biochemical changes are also described as 6H-Syndrome (High FGF23, Hyperphosphaturia, Hypophosphatemia, Hypovitaminosis D, Hypocalcemia and secondary Hyperparathyroidism) causing osteomalacia, bone fractures, muscular weakness and respiratory failure in rare cases [117]. FGF23 further reduces the secretion of EPO and directly enhances erythrocyte apoptosis [266]. This may negatively affect AI pathogenesis, along with an effect of FGF23 on immune activation and a subsequent increased of hepcidin production [266,267,268,269,270,271].

### 5.2. Erythropoiesis-Stimulating Agents

Treatment with recombinant human erythropoiesis stimulating agents (ESA) is a widely used therapy for AI and specifically used in patients with CKD or cancer [1,2]. Beside the initially developed recombinant erythropoietins and the extended half-life product darbapoetin, several Epo biosimilars with different half-life and EpoR affinity are now available [272]. Nevertheless, several studies suggest a more restrictive use of ESAs due to side effects including increased mortality in some specific patient groups. Specifically, a Cochrane meta-analysis showed a decreased overall survival with increased risk for thromboembolic complications, hemorrhage, and hypertension in anemic patients with cancer [273]. Moreover, a recent study demonstrated that ESAs might stimulate tumor growth and progression via the ephrin type-B receptor 4 (EPHB4) that triggers downstream signaling via STAT3 [274]. Determining the tumorous expression of EPHB4 might serve as a screening tool to identify patients with low risk of tumor progression after treatment with ESAs [9]. In addition, ESAs are associated with an increased risk of death or cardiovascular events in both patients with CKD receiving dialysis [275], but also patients not receiving dialysis having a poor initial hematopoietic response [276,277]. Interestingly, functional ID with lower TfS and elevated inflammatory markers are associated with a poor hematopoietic response to ESA treatment [277].

Several mechanisms limit the effectiveness of ESAs in patients with AI. First of all, cytokines not only suppress EPO production, they also suppress EPO-mediated signaling [174] and directly impair erythroid cell proliferation and differentiation [180]. Moreover, EPOR expression is inhibited by FGF23 [268], which is elevated in patients with an activated immune system [267,278,279,280], hyperparathyroidism [281] or hyperphosphatemia [282]. Importantly, ID also negatively impacts on EPO signaling in erythroid cells via its effect on Scribble expression [98], but also blunts erythroid differentiation at later stages [283]. Therefore, treatment strategies enabling a reduction of ESAs dosages by supplementing iron or mobilizing iron via anti-hepcidin therapeutic strategies gained increasing interest over the last years [283].

### 5.3. Blood Transfusions

Blood transfusions are an established and rapid therapy in patients with severe or life-threatening anemia, especially in those with concomitant pathologies including bleeding or chemotherapy induced anemia [1]. Because of the increased risk of allergic reactions, acute hemolytic reactions, acute lung injury and the lack of evidence showing an improvement in outcome, a restrictive blood transfusion strategy is recommended for almost all pathologies [2,284,285]. Actually, two observational studies in anemic HF patients demonstrated that patients receiving blood transfusions had a worse clinical outcome and prognosis [286]. Also in anemic patients with advanced CKD, blood transfusions were shown to cause hyperkalemia and heart failure [287]. Finally, numerous meta-analyses in cancer patients have demonstrated an increased mortality, morbidity and risk for tumor recurrence in patients receiving blood transfusions [255]. Restrictive transfusion protocols seem to be associated with a better outcome [288]. Actually, restrictive use of blood transfusion was shown to be associated with a lower mortality than liberal blood transfusion strategy in critically ill patients [285].

## 6. Novel Therapeutic Principles

Although iron supplementation remains first line therapy for patients with AI and concomitant IDA, new treatment strategies targeting the restricted iron storages in patients with pure AI gained interest in the last years. Mobilization of stored iron from macrophages of the reticuloendothelial system (RES) is probably more suitable than repeated iron supplementation as in the course of advanced inflammation supplemental iron is mainly caught in the RES based on the inhibitory activity of hepcidin on FPN1 mediated iron egress [69,73]. There are two new main therapeutic strategies of rearranging body iron stores: modification (down-regulation) of hepcidin synthesis and function, and stabilization of HIF via inhibition of PHD.

### 6.1. Hepcidin-Modifying Treatments

Hepcidin is recognized as master-regulator of iron metabolism and centrally involved in the pathophysiology of AI. Its effects can be modified by neutralization of hepcidin in the circulation, by targeting hepcidin expression in the liver or by antagonizing hepcidin binding to its receptor, FPN1. (see next chapter).

First studies investigated the effects of hepcidin antibodies on disease progression in mice with AI. The human anti-hepcidin monoclonal antibody Ab12B9m was shown to neutralize hepcidin in vitro and in vivo and to improve anemia in mice when combined with ESA [289,290,291]. Recently a phase I clinical trial investigating effects and safety of the fully humanized monoclonal antibody LY2787106 with a high affinity for hepcidin was tested in cancer patients with anemia. LY2787106 dose-dependently increased serum iron concentrations and TfS within 24 h after administration [292]. Another pharmaceutical approach to hepcidin neutralization is the use of the Spiegelmer^®^ lexaptepid pegol. It binds hepcidin with a high affinity and thus inhibits its biological function [293]. A recent placebo-controlled phase I clinical trial demonstrated that lexaptepid pegol increases serum iron concentrations and TfS in a dose-dependent manner [294] and significantly inhibits hepcidin mediated induction of hypoferremia in healthy humans injected with *Escherichia coli* LPS [295]. In a pilot phase II study in patients with cancer related anemia, lexaptepid pegol increased hemoglobin concentrations and CHr, and decreased sTFR and ferritin concentrations in 5 of 12 patients [296].

Finally, hepcidin effects can be modified by blocking its effector binding site. LY2928057 was recently shown to bind FPN1 thus blocking its interaction with hepcidin and allowing iron efflux. This leads to an increase of serum iron concentrations, TfS and hepcidin with a slower decline of hemoglobin concentrations and a reduction of ferritin concentrations in CKD patients. [297,298] Also, anticalins which are biopharmaceuticals derived from human lipocalins that bind to protein targets, might be effective by mimicking antibody activity [299]. The anticalin PRS-080 decreased hepcidin concentrations with a subsequent increase of serum iron concentrations and TfS in a phase I clinical trial with healthy subjects and CKD patients [300,301].

### 6.2. Modifying Hepcidin Synthetisation by Targeting the BMP/SMAD Pathway

Considering the importance of BMP/SMAD signaling not only in iron-related regulation of hepcidin expression but also in inflammation-related hepcidin expression, pre-clinical studies investigated the effect of BMP/SMAD pathway inhibitors on disease progression of AI [62].

First, inhibition of BMP/SMAD signaling by the BMPR1 (ALK2/ALK3/ALK6) inhibitor dorsomorphin and its derivate LDN-193189 as well as the human recombinant BMP ligand antagonist ALK3-Fc and the soluble hemojuvelin-Fc (HJV-Fc) were shown to reduce hepcidin expression in animal models of AI thus stimulating mobilization of iron from tissue stores and resolving anemia [164,302,303,304,305]. However, dorsomorphin and LDN-193189 also significantly inhibit TGF-𝛽 receptors (TGF𝛽R) at moderate concentrations with the consequence of more interactions [306]. Thus, a new class BMPR1 inhibitor named LJ00328 with a greater selectivity for BMPRs than for TGF𝛽R was developed. LJ00328 was demonstrated to have the greatest potency against ALK2/ALK3 which are the preferentially BMP2/6 receptors for hepcidin induction in the liver [307]. Indeed, LJ000328 significantly repressed hepcidin production in a mice model of iron refractory IDA [308]. Another animal study revealed that the JAK1/2 inhibitor momelotinib (MMB) ameliorates anemia in a rodent AI model by inhibiting ALK2 [309]. Actually, results from a phase 2 study in patients with myelofibrosis showed an improvement of anemia after MMB treatment [310].

In 2011, commercial heparins were shown to efficiently inhibit hepcidin expression in vitro and in vivo by binding and sequestering BMP6, however, with the unfavorable side effect of its anticoagulant activity [311]. Since BMP6 is one of the two main BMP regulators of *HAMP* expression, this led to the development of modified heparins with low anti-coagulant and high hepcidin suppressing activity. Modified over-sulfated (SSLMWH-1) or glycol-split heparins (RO-82) as well as the heparinoid pentosan polysulfate (PPS) exert a similar inhibition of hepcidin expression with iron redistribution concomitant to a reduced anticoagulant activity in vitro and in vivo in mice thus depicting a good alternative [312,313,314]. Recently, a low anticoagulant heparin-iron complex was shown to inhibit hepcidin expression and ameliorate AI in mice as well [315], however, it could not inhibit hepcidin formation upon bacterial infection in mice which was also true for dorsomorphin [316]. Interestingly, loss of BMP6 or HJV represses SMAD related *HAMP* expression in the liver thus causing increased extrahepatic iron loading [317]. This demonstrates the complex regulator networks affecting hepcidin expression suggesting that pharmacological hepcidin repression must be critically analyzed and indicated.

More recently, a fully human anti-BMP6 antibody (KY1070) was shown to counteract the hepcidin-driven iron limitation for erythropoiesis in two-different, well-established, AI rodent models. In addition, combination with darbepoetin alfa further improved the erythroid response when compared to monotherapy [283]. Another study in CKD patients showed that LY3113493 specifically blocked binding of BMP6 to its receptor with an increase of serum iron and TfS and decrease of hepcidin levels [297].

However, BMP signaling is critically involved in normal cardiovascular structure and function, bone homeostasis, and progression of specific cancers [318] which limits the therapeutic approach of broadly effective BMP signaling inhibitors for specific diseases such as AI. Unfortunately, clinical trials with human patients investigating the effects of BMP signaling inhibition in patients with AI are lacking by now.

### 6.3. Stabilization of Hypoxia-Inducible Factor

Another new promising therapy strategy is the inhibition of prolyl hydroxylase, which stabilizes hypoxia-inducible factor thus promoting erythropoietin production, increasing intestinal iron uptake and mobilization of iron stores [319]. Oral hypoxia-inducible factor prolyl hydroxylase inhibitors (HIF-PHI) including roxadustat, vadadustat, desidustat, daprodustat and molidustat were shown to significantly increase hemoglobin levels and total iron binding capacity concomitant to an decrease in hepcidin and ferritin levels in patients with anemia and chronic kidney disease [320]. Moreover, several preclinical studies have suggested a potential cardiovascular benefit due to direct actions of HIF activation in myocardial infarction, cardiac remodeling and atherosclerosis as well as amelioration of glucose and lipid metabolism [321]. Actually, roxadustat was shown to reduce the average total cholesterol regardless of lipid-lowering treatments [322,323,324].

In a recent meta-analysis by Chen and coworkers including 13,146 patients, HIF-PHI were well tolerated during long-term use with comparable risk of serious adverse events compared to patients treated only with ESAs. However, HIF-PHI treatment was associated with a slightly increased risk for thrombosis events when compared to patients treated only with ESAs [320]. Several phase 3 trials further evaluating the efficacy and safety of HIF-PHI in CKD patients including the long-term cardiovascular risk are currently ongoing. Interestingly, roxadustat also raised hemoglobin levels in CKD patients with immune activation [323,324,325], which suggests that HIF-PHI might be a possible treatment strategy for anemia in patients with other chronic inflammatory disease, a notion that needs to be experimentally and clinically verified.

## 7. Nutrition in the Treatment of Anemia of Inflammation

Several nutrients including vitamins, amino acids and iron are essential for hemoglobin synthesis and erythropoiesis [326]. Especially vitamin B9 (folic acid) and vitamin B12 (cobalamin) are indispensable for heme synthesis and erythropoiesis. Deficiency of these two vitamins results in (hemolytic) anemia or further aggravates anemia in patients with inflammatory disorders [327]. In patients with AI and concomitant vitamin B9 or B12 deficiency, these vitamins should be supplemented [2]. Interestingly, the synthetic vitamin B1 (thiamine) derivative fursultiamine was shown to inhibit hepcidin-induced Fpn1 internalization thus promoting cellular iron export in vitro [328]. In a mice model of intracerebral hemorrhage, fursultiamine was demonstrated to improve brain iron efflux and reduce oxidative brain injury aggravated by hepcidin [329].

Vitamin D supplementation was shown to inhibit hepcidin expression in patients with CKD and healthy adults [330,331] with a significant increase of hemoglobin levels in critically ill patients [332]. Actually, vitamin D response elements (VDREs) are found in the promoter region of HAMP and have a suppressing effect [330]. Vitamin D receptor signaling further has anti-inflammatory effects, which may positively affect the course of an underlying chronic inflammatory disease [333,334]. In fact, vitamin D plays a crucial role in the modulation of innate and adaptive immune response [335] and can induce a shift from a proinflammatory to a more tolerogenic immune status with diverse effects on T cell subtypes: e.g., it suppresses Th1-cell proliferation, differentiation and cytokine production [336,337]. Furthermore, also Th2-cells [338] and Treg-cells are induced [339], while Th17-mediated autoimmunity can be ameliorated by 1,25-dihydroxyvitamin D_3_ [340]. As chronic diseases often go along with vitamin D deficiency [341] and low vitamin D levels are correlated with higher immune activation markers like neopterin, e.g., in CAD patients [342], vitamin D supplementation of patients might be very effective to down-regulate underlying inflammatory processes.

Dietary vitamin C (ascorbate) provides reducing capacity for Fe^3+^ reduction thus enhancing the activity of the enzyme ferric reductase Dcytb and increasing iron absorption [241]. Vitamin C supplementation must be considered as additional treatment especially in patients with concomitant IDA. In addition, vitamin C was demonstrated to increase ferritin concentrations and stimulate transferrin-dependent iron uptake [343,344,345]. Finally, vitamin C directly inhibits hepcidin production and enhances EPOR expression in human liver carcinoma cells [346] thus exerting an additional effect on erythropoiesis in combination with EPO [347]. Vitamin C is also one of the most potent antioxidants and plays an important role for immune function, e.g., it can enhance differentiation and proliferation of B- and T-cells [348]. As higher vitamin C intake and plasma levels were inversely associated with C-reactive protein concentrations [349], and lower vitamin C and E concentrations coincide with higher neopterin concentrations in patients with CAD [350], vitamin C appears to have anti-inflammatory properties [349], which might be beneficial for patients with chronic immune activation. As vitamin C is also a co-factor for the asparagyl and prolyl hydroxylases required for the downregulation of HIF-1α [351], decreased vitamin C availability might also impact erythropoietin formation.

The polyphenol curcumin was also shown to reduce pro-inflammatory cytokine production and directly decrease hepcidin transcription in vitro and in vivo [352,353]. Finally, the Nrf2 activator sulforaphane (SFN), which is a broccoli-derived isothiocyanate (ITC) was shown to counteract the inflammatory induced hepcidin expression with consequent repression of Fpn1 expression in murine macrophages and human cancer cells [146,354].

Finally, diet supplementation with the iron-binding protein lactoferrin seems to be advantageous in the prophylaxis and treatment of mild iron deficiency but may also exert anti-microbial activities [355]. Apart from direct pathogen destruction lactoferrin also regulates cells of innate and adaptive immune system by stimulating the secretion of anti-infectious and anti-tumor cytokines [356,357]. Lactoferrin changes the macrophage phenotype from pro-inflammatory to anti-inflammatory thus decreasing IL-6 and hepcidin expression, while increasing FPN1 and ceruloplasmin expression thereby promoting iron egress from macrophages [358,359].

## 8. Conclusions

Anemia of inflammation (AI) is a very frequent clinical condition affecting more than a billion people worldwide. A deep understanding of the underlying pathophysiology along with the correct diagnosis of anemia of inflammation and with identification of aggravating factors for anemia severity is vital for selecting the most appropriate therapy. Apart from established treatments including iron supplementation, erythropoiesis stimulating agents or blood transfusions, new therapeutic options, including modulators of hepcidin function or hypoxia inducible factors prolyl hydroxylase inhibitors were identified and evaluated mostly in pre-clinical studies over the last years. However, some of them have entered into clinical trials and those results concerning anemia correction in different inflammatory disorders are awaited.
